# A Novel ZAP-70 Dependent FRET Based Biosensor Reveals Kinase Activity at both the Immunological Synapse and the Antisynapse

**DOI:** 10.1371/journal.pone.0001521

**Published:** 2008-01-30

**Authors:** Clotilde Randriamampita, Pierre Mouchacca, Bernard Malissen, Didier Marguet, Alain Trautmann, Annemarie Coffman Lellouch

**Affiliations:** 1 Institut Cochin, Université Paris Descartes, CNRS (UMR 8104), Paris, France; 2 Inserm, U567, Paris, France; 3 Centre d'Immunologie de Marseille-Luminy, Université de la Méditerranée, Marseille, France; 4 Inserm, U631, Marseille, France; 5 CNRS, UMR6102, Marseille, France; New York University School of Medicine, United States of America

## Abstract

Many hypotheses attempting to explain the speed and sensitivity with which a T-cell discriminates the antigens it encounters include a notion of relative spatial and temporal control of particular biochemical steps involved in the process. An essential step in T-cell receptor (TCR) mediated signalling is the activation of the protein tyrosine kinase ZAP-70. ZAP-70 is recruited to the TCR upon receptor engagement and, once activated, is responsible for the phosphorylation of the protein adaptor, Linker for Activation of T-cells, or LAT. LAT phosphorylation results in the recruitment of a signalosome including PLCγ1, Grb2/SOS, GADS and SLP-76. In order to examine the real time spatial and temporal evolution of ZAP-70 activity following TCR engagement in the immune synapse, we have developed ROZA, a novel FRET-based biosensor whose function is dependent upon ZAP-70 activity. This new probe not only provides a measurement of the kinetics of ZAP-70 activity, but also reveals the subcellular localization of the activity as well. Unexpectedly, ZAP-70 dependent FRET was observed not only at the T-cell -APC interface, but also at the opposite pole of the cell or “antisynapse”.

## Introduction

The question of how the signalling machinery downstream of the T-cell Receptor (TCR) rapidly integrates the binding information resulting from the encounter of a T-lymphocyte with an antigen presenting cell (APC) is central to our understanding of the adaptive immune response. Many hypotheses attempting to explain the speed and sensitivity with which a T-cell discriminates the antigens it encounters, include a notion of relative spatial and temporal control of particular biochemical steps involved in the process (reviewed by Burroughs and van der Merwe, [1]). Since Kupfer and colleagues first described a non-homogeneous distribution of signalling molecules at the lymphocyte-APC interface, fluorescence microscopy techniques used in conjunction with labelled antibodies or autofluorescent protein fusion constructs have become indispensable tools in mapping the redistribution or translocation of signalling molecules in the immune synapse during T-cell activation [2]–[4]. Such techniques provide dynamic information about cellular processes with subcellular resolution that has otherwise been inaccessible to more classical biochemical techniques (e.g. western blots) traditionally employed to study signal transduction. However, despite the obvious power of imaging, one can infer limited information about enzyme activity or dynamic posttranslational modifications such as phosphorylation, from the translocation or redistribution of a given molecule.

TCR engagement leads rapidly to the activation of the Syk family tyrosine kinase ZAP-70. ZAP-70 activation requires both Src family kinase dependent phosphorylation of tyrosine residues in its kinase domain and a conformational switch induced by the binding of its dual SH2 domains to phosphorylated motifs, such as those found in the cytoplasmic tail of the TCR CD3ζ chain [5]. Once activated, ZAP-70 phosphorylates multiple tyrosine residues on the adaptor protein LAT, resulting in the assembly of a signalling complex which includes PLCγ1, Grb2/SOS, GADS and SLP-76 [6]. The regulation of ZAP-70 activity is a complex yet key step in TCR mediated signal transduction. ZAP-70 is proposed to be involved in feed-back loops required to set a threshold for TCR sensitivity [7], [8].

Numerous studies employing ZAP-70 GFP fusion proteins have demonstrated that ZAP-70 is rapidly recruited to the cell membrane upon TCR engagement [9]–[11]. Interestingly, in an early study in which fixed immune synapse samples were probed with phospho-epitope specific antibodies, it was shown that phospho-ZAP-70 had a shorter lifetime at the synapse than the global ZAP-70 population [12] suggesting that the dynamics of ZAP-70 activity and that of ZAP-70 membrane recruitment are not necessarily equivalent. Elegant strategies have since been described for creating genetically encodable biosensors which may undergo a phosphorylation dependent change in FRET ratio and therefore can be used to directly visualize the temporal and spatial evolution of a given protein tyrosine kinase activity using fluorescence video microscopy [13], [14]. In order to examine the dynamic behavior of ZAP-70 activity in the immunological synapse more directly, we present here the creation of a novel biosensor, ROZA (for **R**eporter **O**f **Z**AP-70 **A**ctivity) which is comprised of elements of mouse Grb2, mouse LAT and the CFP-YFP pair of fluorescent donor and acceptor. Here we report the use of ROZA expressing cells to directly visualize ZAP-70 dependent phosphorylation in T-cell lines and primary human lymphocytes with subcellular resolution during the formation of an immunological synapse. Furthermore, we observe that ZAP-70 dependent activity revealed by ROZA displays a bipolar distribution, appearing at times at the pole opposite that of the lymphocyte-APC contact. To our knowledge, ROZA is the first biosensor engineered for a TCR dependent tyrosine kinase activity and as such represents a key step forward towards bringing the wealth of biochemical information available about TCR mediated signal transduction into the realm of live cell microscopy.

## Results and Discussion

### Design of the FRET probe ROZA

**Figure 1:**
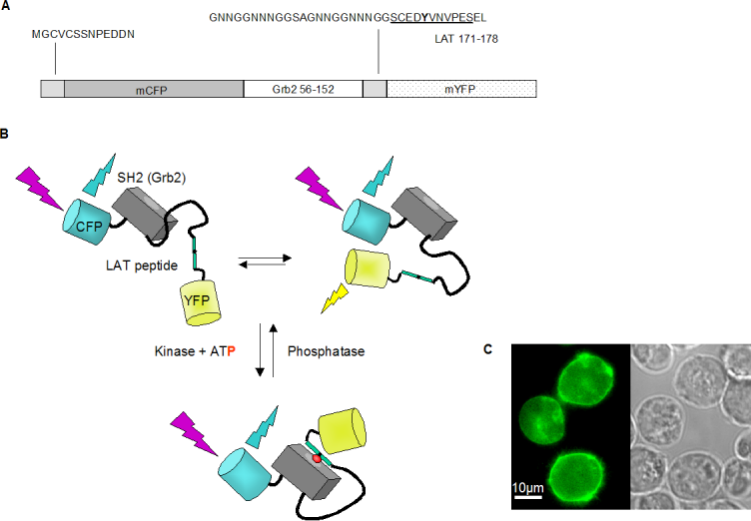
Structure and subcellular localization of ROZA. (A) Scheme of the ROZA sequence including the N-terminal residues from p56 Lck, the linker sequence and residues 171-178 of LAT (underlined) surrounding Y175 (in bold). (B) Model showing the schematic structure of ROZA (without its membrane anchor), and illustrating that, in the absence of ZAP-70 activity, ROZA may adopt several conformations, some of them allowing FRET. Following phosphorylation of the LAT based sequence by ZAP-70, ROZA adopts a constrained conformation incompatible with FRET. (C) In Jurkat T-cells transiently transfected with ROZA, the probe is mainly located at the plasma membrane as visualized by CFP fluorescence.

As previously described [13], [14], a ratiometric FRET based probe for a tyrosine kinase activity can be prepared by fusing a peptide sequence encoding a known substrate sequence for the targeted kinase with its cognate phospho-tyrosine binding domain (e.g. a Src homology (SH) 2 domain). When assembled with the appropriate linker sequences and flanked by compatible donor and acceptor autofluorescent proteins, such an engineered protein can undergo a phosphorylation dependent change in its FRET signal, presumably due to a phosphorylation dependent intramolecular binding event which changes the relative distance and/or orientation of the autofluorescent protein domains (See Figure 1B). In order to create a ZAP-70 specific probe, we targeted peptide sequences derived from mouse LAT encompassing tyrosine residues Y132, Y175 or Y195, known to be substrates for ZAP-70 [15]. The peptides were fused to their respective SH2 domain binding partners such as the N-terminal SH2 domain of mouse PLCγ1 for Y132 and the SH2 domain of mouse Grb2 for Y175and Y195 [16]. ROZA variants were produced in which the substrate peptide, the SH2 binding partner as well as the length and composition of the interdomain linkers were varied. Constructs were screened for their expression levels in Jurkat J-Tag cells and their ability to generate a phosphorylation dependent change in FRET signal upon pervanadate (PV) and anti-CD3 stimulation. The sequence resulting in the most significant phosphorylation dependent FRET change (see Fig. 1A) was used for all subsequent experiments. In order to facilitate the encounter of active ZAP-70 with the substrate probe, ROZA was targeted to the plasma membrane by incorporating the 13 *N*-terminal residues of mouse Lck. As shown in Fig. 1C, the protein is indeed largely restricted to the membrane of Jurkat T-cells. A variant was also prepared incorporating the *N*-terminal 35 residues of LAT, encompassing the transmembrane domain and palmitoylation sites, however this anchor led to a largely vesicular probe expression (data not shown).

**Figure 2:**
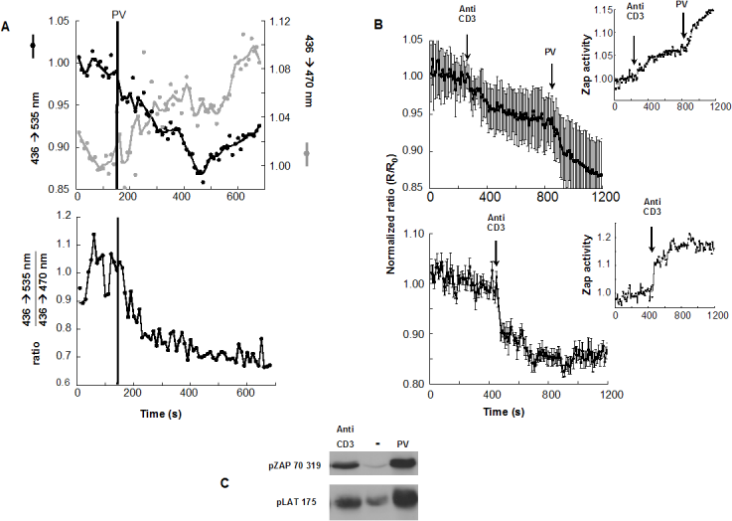
(A) In ROZA-expressing Jurkat T-cells, PV induces changes in the 436→535 and in the 436→470 signals (top) and in their ratio (bottom). Traces correspond to the mean of 3 different T-cells. (B) In Jurkat T-cells (top), anti-CD3 induces a partial activation of ZAP-70 that can be completed by PV. In peripheral blood T-cells (bottom), anti-CD3 triggers a large and rapid FRET decrease. Inset: ZAP-70 activity is shown as the inverse of the FRET signals. Traces correspond to the average of 8–17 individual cells. (C) In Jurkat T-cells stably expressing ROZA and stimulated with PV or anti-CD3, ZAP-70 (site 319) as well as endogenous LAT (site 175) are phosphorylated after a 2 minute stimulation.

Stimulation of ROZA-expressing Jurkat T-cells with pervanadate (PV), a phosphatase inhibitor that unveils constitutive tyrosine kinase activity, triggered a simultaneous increase of the 436→470 nm signal and a decrease of the 436→535 nm signal (Fig. 2A upper panel). Stimulation thus triggers a decrease of the 535 nm/470 nm ratio, i.e., of the FRET signal (Fig. 2A lower panel). Changes in FRET signals could also be detected in Jurkat T-cells (Fig. 2B upper panel) or human PBT (Fig. 2B lower panel) after stimulation with anti-CD3. The ROZA expressing Jurkat T-cells were similarly stimulated by either anti-CD3 or PV and subsequently lysed and subjected to western blot analysis. Both phosphorylated forms of ZAP-70 and endogenous LAT where readily detectable 2 minutes (120 seconds) after stimulation, confirming that ZAP-70 is indeed activated under these conditions in the ROZA expressing cells (Fig. 2C). The stimulation dependent decrease in FRET is compatible with a model in which the autofluorescent proteins of the non-phosphorylated form of the probe have a relative distance or orientation which allows FRET, whereas the phosphorylated form of the probe assumes a conformation less compatible with FRET. Other FRET based kinase activity probes described in the literature function with a phosphorylation dependent decrease in FRET capacity [17], [18]. The most relevant example is the second generation Src kinase reporter [18], which like ROZA incorporates an SH2 phospho-tyrosine binding domain, and also undergoes a phosphorylation dependent decrease in FRET. The authors postulate that the close positioning of the N and C termini of the SH2 domain would allow a relative proximity of the linked autofluorescent protein domains and thus FRET in the probe's basal state. This relative proximity would be disrupted during the phosphorylation dependent intramolecular binding event and the FRET ratio would decrease. The N and C termini of the SH2 domain of Grb2 are also found on the same face of the domain in its crystal structure which is consistent with the FRET decrease observed in ROZA [19]. Fig 1B illustrates such a model, taking into account the possibility that in the basal state, the probe may adopt several conformations. In the following figures, ZAP-70 activity is arbitrarily expressed as the inverse of FRET ratio (Fig. 2B inset). Using this representation a FRET decrease corresponds to an increase in net ZAP-70 dependent activity.

### Both ROZA FRET and phosphorylation are ZAP-70 dependent

**Figure 3:**
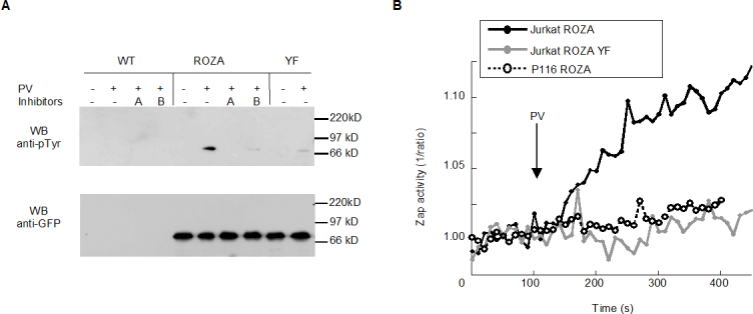
ROZA is specific for ZAP-70. (A) WT J77cl20 or cells transiently expressing either ROZA or ROZA YF (5×10^6^ cells/point) were preincubated for 30 minutes with inhibitors (A. 10 µM PP2 or B 200 µM piceatannol) or medium alone, followed by a 2 minute activation with 5 mM freshly prepared PV. ROZA was immunoprecpitated with anti-GFP antibody. Upper panel: anti-phosphoTyr (4G10) western blot. Lower panel: anti-GFP western blot. (B) PV induces an increase in ZAP-70 activity in Jurkat T-cells expressing ROZA. No change in FRET ratio was observed in ZAP-70-deficient Jurkat T-cells (P116) cells expressing ROZA or in normal Jurkat T-cells transfected with the mutated probe (YF).

In order to establish that the observed FRET changes were due to a ZAP-70-dependent phosphorylation of the probe, Jurkat T-cells transiently expressing ROZA were stimulated, lysed, and subsequently subjected to anti-phosphotyrosine immunoprecipitation and anti-GFP immunoblotting (Fig. 3A). The resulting immunoblots of the transfected Jurkat T-cell samples displayed an activation-dependent phosphorylated species with the expected molecular weight of the ROZA probe (69 kD). Pre-treatment of transfected cells with the Syk family inhibitor piceatannol, or with the Src kinase inhibitor PP2, resulted in inhibition of ROZA phosphorylation. Similarly, mutation of the probe's target tyrosine residue LAT 175 to phenylalanine resulted in significant inhibition of the probe phosphorylation (Fig. 3A upper gel). Stripping and reblotting with an anti-GFP antibody confirmed that the most prevalent GFP containing species had a molecular weight consistent with the full length ROZA (Fig. 3A lower gel).

Additional specificity tests were performed by imaging. As expected, the FRET changes triggered by anti-CD3 stimulation were strongly inhibited in cells pretreated with PP2 for 30 minutes before stimulation (data not shown). The strong fluorescence of piceatannol precluded its use in FRET experiments. Cells expressing the YF175 ROZA mutant also displayed no significant change in the FRET signal upon PV stimulation confirming that phosphorylation of the LAT sequence found within ROZA was necessary for the FRET ratio change. Finally, in ZAP-70-deficient Jurkat T-cells, P116, transfected with ROZA, no significant change in the FRET ratio was observed upon stimulation (Fig. 3B). This clearly shows that the CED**Y**VNV tyrosine phosphorylation motif present in the probe cannot be phosphorylated by Src kinases in the absence of ZAP-70.

### ZAP-70 activity can be visualized in the immunological synapse

**Figure 4:**
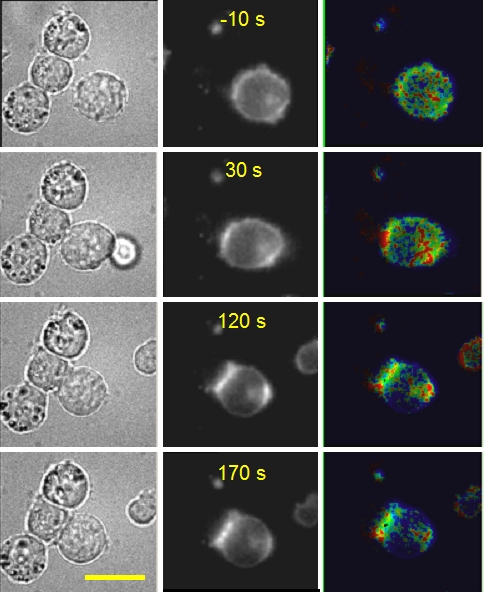
Synaptic and antisynaptic activation of ROZA. Sequence of events observed upon interaction of a ROZA-expressing Jurkat T-cell with superantigen-loaded Raji B cells. Left: transmitted light images; center: subcellular ROZA localization; right: ZAP-70-dependent activity in false colours, 1/R ranging from 1.25 (blue) to 1.7 (red). Time zero corresponds to the initial contact, as detected in transmitted light images. The bar corresponds to 10 microns.

In order to validate the utility of ROZA for examining the evolution and subcellular distribution of ZAP-70 dependent phosphorylation during a cell-cell activation event, we followed the formation of conjugates between Jurkat clones stably expressing ROZA and Raji B cells loaded with superantigen under 40X magnification. Rapidly after conjugate formation, a synaptic clustering of the probe was observed. Such a recruitment could be due to the fact that the probe is anchored to the membrane through a palmitoylated and myristylated N-terminal sequence derived from the Src kinase Lck, that presumably targets ROZA into lipid rafts [20]. Accumulation of ROZA at the immunological synapse was generally quite marked (Fig. 4). However ZAP-70 dependent phosphorylation as revealed by the ROZA FRET change was detected before the accumulation of the probe at the synapse (data not shown). The ROZA signal was triggered and reached its maximal value within a minute of contact formation, consistent with literature reports of global LAT phosphorylation kinetics [21]. Unexpectedly, ROZA accumulation and FRET evolution was also frequently observed at the cell pole opposite to the synapse, or the “antisynapse”. In some cases, ROZA FRET and accumulation was observed first at the synapse whereas in other cases it was observed first at the antisynapse. Fig. 4 illustrates a case where both poles appeared simultaneously. Antisynaptic localisation such as we observed with ROZA has previously been observed with other synaptic molecules such as CD3, or PIP3 [22], [23]. However little information is currently available about either the dynamics or the mechanisms underlying this phenomenon.

### Speculation: Is double signalosome formation a simple biological pattern?

The appearance of a double signalosome could be considered as a simple case of biological pattern formation. In the theory of biological pattern formation initially proposed by Turing and later revisited by others, a pattern-forming reaction uses a combination of self-enhancing local activator and a long-range inhibitor triggered by the activator, and may create a dead zone for activator accumulation around the initial accumulation [24], [25]. Similarly, we propose that in T-cells, the initial synaptic contact may trigger the production of an auto-catalytic activating element capable of forming a signalosome. The concomitant production of faster diffusing inhibitory elements initially restricts the formation of the signalosome to the synapse. However if the diffusing inhibitory elements become diluted at the antipodal region of the cell, activator accumulation may occur at the anti-synapse. The formation of a second signalosome may be facilitated by a cell asymmetry created by the first signalosome, or by a diffusing activator, or by cell polarization associated with cell movement.

## Materials and Methods

### Cells and reagents

JTAG, J77 clone 20 and Raji B cells were kinds gifts of Georges Bismuth (Institut Cochin, Paris, France); ZAP-70 deficient Jurkat T-cells P116 were a kind gift of Claire Hivroz (Institut Curie, Paris, France). Human T lymphocytes (PBT) were isolated from blood donors by Ficoll density gradient centrifugation, followed by negative depletion on magnetic beads (T-cell negative isolation kit; Becton Dickinson). Cells were cultivated in RPMI 1640 supplemented with 10% FCS. Superantigen was a mix of recombinant staphylococcal enterotoxin E, staphylococcal enterotoxin A, staphylococcal enterotoxin B, and staphylococcal enterotoxin C3 (Toxin Technology). Raji B cells were loaded for 30 min at 37°C in RPMI with superantigen at a final concentration of 200 ng/ml. All PCR amplifications were performed using Taq polymerase (Invitrogen). Sequencing was performed by MWG-Biotech AG (Ebersberg, Germany). DNA modifying enzymes were obtained from Invitrogen. Synthetic nucleotides were obtained from Sigma-Genosys, Ltd. (Cambridge, U.K.). Plasmids encoding mCFP and mYFP were kindly provided by R.Y Tsien (UCSD, USA). Kinase inhibitors PP2 and piceatannol were from Calbiochem. Mouse anti-human anti-CD3 (UCHT1) was from BD Pharmingen. HRP coupled goat anti-mouse antibody was from Immunotech SA (Marseille France). Anti-phospho-tyrosine antibody 4G10 ascites was a kind gift of Hai-Tao He, (Marseille, France). Anti-GFP antibody was from Abgene (U.K.) Anti phospho-ZAP70 319 and anti-phospho LAT171 were from Cell Signalling (USA).

### Construction of ROZA

The sequence encoding ROZA was constructed from three fragments. A CFP fragment was created by PCR amplification of mCFP residues 1-228 using a coding primer incorporating a *HindIII* site, a kozak sequence, and the 13 *N*-terminal residues of mouse Lck and a non-coding primer incorporating a *SphI* restriction site. Mouse Grb2 (residues 56-152) cDNA was amplified using a coding primer incorporating a *SphI* site and a non-coding primer which incorporated the linker sequence, mouse LAT residues 171-178 and a *SacI* site. The specific amino acids created were as shown in Fig. 1A. A YFP fragment was created by amplifying full length mYFP with a coding primer including a *SacI* site and a non-coding primer incorporating a stop codon and an *EcoRI* site. The three fragments were ligated simultaneously into the *HindIII/EcoRI* sites of pCDNA3.1. The Tyr to Phe mutation of the LAT tyrosine 175 present in central fragment of the ROZA sequence was created using nested amplifications of the Grb2-linker LAT sequence. The complete nucleotide sequence encoding this structure has been deposited in Genebank (accession number EU035753).

### Cell transfections

Jurkat T-cells and PBT were transfected by Amaxa nucleofection with solution V/ Program G-10 or S-18 and human T solution / Program U-14, respectively. Typically 5×10^6^ cells were transfected with 5 µgs plasmid DNA. Cells were used 24–48 hours after nucleofection. Stable clones of the Jurkat J77 cl20 expressing ROZA were established first sorting the YFP positive cells with a Becton Dickenson FACSAria cell sorter followed by cloning by limiting dilution in the presence of 1.5 mg/ml G418 (Invitrogen).

### Imaging

Fluorescence acquisition was performed with a Nikon TE2000 equipped with cooled CCD camera (Cascade, Princeton Instruments). Three images were acquired every 10s: visible, excitation at 435 nm and emission successively at 530 nm (FRET) and 470 nm (CFP). The ratio R = FRET/CFP that gives an estimation of the inverse of ZAP-70 activity, was calculated with MetaFluor (Roper Scientific) after background subtraction. The following filters were used, all from Chroma (Rockingham, US), except when otherwise mentioned. For CFP excitation and emission: 436±5 nm→470±15 nm. For YFP: 500±10 nm→535±20 nm.

### Immunoprecipitation and immunoblotting

ROZA expressing cells were cultured at densities less than 0.5×10^6^ cells per ml, recovered, rinsed once in serum-free medium and suspended at 5×10^6^ cells per point in RPMI supplemented with 10 mM HEPES with or without kinase inhibitors. Cells were stimulated with the pervanadate or antibody for the indicated time, briefly spun, and resuspended in ice cold NP-40 lysis buffer (50 mM Tris pH 7.5, 150 mM NaCl, 1% NP-40, 1mM PMSF, 0.4 mM sodium orthovanadate, and Complete protease inhibitor Mix (Roche) as per manufacturers instructions) at 4°C for 20 minutes. In the indicated experiment, post nuclear supernatants were subjected to immunoprecipitation with an anti-GFP antibody to pull down the probe. Immunoprecipitates were subjected to SDS-PAGE transferred to PVDF membrane. Membranes were probed with indicated antibodies as per manufacturers recommandations for each specific antibody followed by HRP conjugated secondary antibody (Immunotech SA, Marseille, France). Images were revealed using ECL plus (Amersham).
